# Systemic Inflammation and Cardio-Renal Organ Damage Biomarkers in Middle Age Are Associated With Physical Capability Up to 9 Years Later: Findings From a British Birth Cohort Study

**DOI:** 10.1161/CIRCULATIONAHA.118.037332

**Published:** 2019-01-22

**Authors:** Diana Kuh, Rachel Cooper, Naveed Sattar, Paul Welsh, Rebecca Hardy, Yoav Ben-Shlomo

**Affiliations:** 1 MRC Unit for Lifelong Health and Ageing, University College London, UK (D.K., R.C., R.H.).; 2 Institute of Cardiovascular and Medical Sciences, British Heart Foundation Glasgow Cardiovascular Research Centre, University of Glasgow, UK (N.S., P.W.).; 3 Population Health Sciences, University of Bristol, UK (Y.B-S.).

**Keywords:** aging, cohort studies, cystatin C, inflammation, natriuretic peptides

## Abstract

**Background:**

Physical capability, a key component of healthy aging, is associated with cardiovascular and other risk factors across life. We investigated whether midlife biomarkers of heart and kidney damage capturing the cumulative impact of long-term adverse exposures were associated with the level and decline in physical capability over 9 years of follow-up, taking account of systemic inflammatory biomarkers and conventional cardiovascular risk factors.

**Methods:**

We used data on 1736 men and women from the oldest British birth cohort study with walking speed, chair rise speed, balance time, and grip strength assessed at ages 60 to 64 and 69 years. We tested associations between logged and standardized measures of cystatin C, NT-proBNP (N-terminal pro-B-type natriuretic peptide), interleukin (IL)-6, and E-selectin at age 60 to 64 years with performance at age 69 years, adjusting for sex, height, and body mass index; then for performance at age 60 to 64 years. These biomarkers were mutually adjusted, and additionally adjusted for cardiovascular risk factors (pulse pressure, total/high density lipoprotein cholesterol, glycosylated hemoglobin), diabetes mellitus, cardiovascular and kidney disease, smoking status, and lifetime socioeconomic position.

**Results:**

Cystatin C, NT-proBNP, and IL-6 (but not E-selectin) were inversely associated with all outcomes, adjusted for sex, height, and body mass index. For example, a 1-SD increase in logged NT-proBNP was associated with weaker grip (−0.63 kg, 95% CI, −0.99 to −0.28); the equivalent association for cystatin C was −0.60 kg (95% CI, −0.94 to −0.25) and for IL-6 was −0.76 kg (95% CI, −1.11 to −0.41). Most associations remained, albeit attenuated, after adjustment for previous performance and mutual adjustment of the biomarkers. NT-proBNP and IL-6 (but not cystatin C) were more strongly associated with the outcomes than many of the conventional risk factors after mutual adjustment.

**Conclusions:**

Higher levels of NT-proBNP may identify those in midlife at risk of accelerated physical decline. Before considering the use of NT-proBNP for risk stratification, further research should untangle whether these associations exist because the biomarker is an integrated measure of cumulative exposures to relevant stressors across life, or whether it is marking additional risk pathways. Randomized trials to reduce the rate of decline in physical capability or delay incident disability could benefit from including middle-aged adults and adding NT-proBNP and IL-6 as intermediate outcomes.

## Abstract 2

Supplemental Digital Content is available in the text.

**Clinical Perspective**

**What Is New?**

- Lower levels of NT-proBNP (N-terminal pro-B-type natriuretic peptide) and interleukin-6 in middle-aged adults were independently associated with better physical capability (a key component of healthy aging) up to 9 years later.
- Such associations were meaningfully stronger than those observed for conventional risk markers including lipids, blood pressure, and glycemia and were not explained by the onset of cardiovascular and kidney disease or diabetes mellitus.

**What Are the Clinical Implications?**

- Elevated NT-proBNP and interleukin-6 in midlife could help identify (and thereby target) individuals set to have poor physical capability as they age.
- Such findings may relate in part to such biomarkers capturing early end-organ damage, or cumulative stressor pathways that lead to physical decline.
- Future trials targeting improvements in physical capability should include middle-aged as well as older adults and use measurements of cardio-renal biomarkers as intermediate outcomes.

**Editorial, see p 2000**

Healthy biological aging can be characterized by the following 3 domains: survival to old age, delay in onset of chronic disease and disability, and optimal physical and cognitive capability for the maximal period of time.^1^ Physical capability, the capacity to undertake the physical tasks of daily living, can be assessed using simple objective measures such as gait speed, chair rise speed, balance time, and grip strength. Poor performance on these measures has been consistently shown to be associated with subsequent hospitalization, morbidity, disability, and mortality.^2–5^ Life course trajectories for these measures show a developmental stage of increased performance followed by a plateau and then a decline phase, although there is marked interindividual variability.^6^

A review of potentially modifiable risk factors for physical capability highlighted poor lifetime socioeconomic conditions, obesity, and smoking, all known risk factors for cardiovascular disease and other chronic conditions.^6^ Similarly, higher blood pressure, diabetes mellitus, and ischemic heart disease risk factors have also been shown to be associated with worse later physical capability in a number of cohort studies.^7,8^ The American Heart Association has recently stated that functional capacity should be prioritized in older adults living with cardiovascular disease, and recommended indicators such as strength and balance.^9^ Whereas most risk factors have been measured in mid- or later life, there is growing evidence that factors from earlier life, including poorer growth and development, as well as adverse lifetime socioeconomic conditions have cumulative long-term negative associations with physical capability, especially muscle strength.^10–14^

A variety of different biological markers measured in the blood capture domains of current health status with relevance to long-term risks. For example, systemic inflammatory markers are nonspecific and react to acute exposures such as infection, and chronically higher levels appear to be the consequence of processes or risk pathways such as obesity, which increase the chances of atherosclerosis. Ideally, we should examine organ damage using biomarkers that capture the cumulative impact of long-term adverse exposures that may impact the level and decline in physical capability. Studies in midlife would be particularly useful to see whether those most at risk of decline could be detected early. There is evidence that cystatin C levels (and their changes with age), a potentially better measure of renal dysfunction than creatinine based measures,^15^ are associated with physical capability in older adults.^16,17^ NT-proBNP (N-terminal pro-B-type natriuretic peptide) is a marker of cardiac stress or damage that is associated with cardiovascular disease and heart failure risk across its entire range of measurement,^18–21^ and with physical activity,^22,23^ but to our knowledge has not been examined in relation to objective measures of physical capability.

Using data from the oldest British birth cohort study, we tested the hypothesis that markers of composite organ damage/stress (cystatin C and NT-proBNP) measured at age 60 to 64 years would show associations with physical capability at age 69 years and the change in physical capability, even after taking account more systemic biomarkers of inflammation/vascular dysfunction (interleukin [IL]-6, E-selectin), and cardiometabolic and lifetime sociobehavioral risk factors. We further hypothesized that these measures of specific organ damage/stress could show stronger associations than those reflecting less specific systemic exposures.

## Methods

The Medical Research Council (MRC) NSHD (National Survey of Health and Development) is a sample of 5362 males and females born in England, Scotland, and Wales in 1 week in March 1946 and followed up since. The 24th data collection was conducted between 2014 and 2015 when study members were aged 68 to 69 years.^24^ At age 69 years, after a postal questionnaire at age 68 years, study members still alive and with a known current address in mainland Britain (n=2698) were invited to have a home visit; 2149 (79.7%) completed a visit and a further 55 (2.0%) completed a postal questionnaire instead. Of the original cohort, 1026 (19.1%) had died, 578 (10.8%) were living abroad, 22 (0.4%) asked for their participation to be restricted to postal contacts, 621 (11.6%) had previously withdrawn from the study, and 417 (7.8%) had been lost to follow-up. Ethical approval for the most recent visit was given by Queen Square Research Ethics Committee (14/LO/1073) and Scotland A Research Ethics Committee (14/SS/1009). Written informed consent was provided by participants for each visit.

### Physical Capability Outcomes

At age 69 years, physical capability was assessed using 4 objective measures: grip strength, chair rise speed, walking speed, and standing balance time. Trained nurses conducted these tests using standardized protocols as summarized here. Grip strength (kilograms) was measured isometrically using a Jamar Plus+ Digital Hand dynamometer. Two values were recorded for each hand while the participant was seated, and the highest value achieved was used in analyses. Chair rise time was measured, using a stopwatch, as the time taken to rise from a sitting to a standing position with straight back and legs and then sit down again 10 complete times as fast as possible. If the participant began the test but was unable to perform all 10 rises (n=26), the number of rises completed was recorded. To take account of differences in the number of rises completed and for high scores to indicate good performance, chair rise speed (stands per minute) was calculated by dividing the number of completed rises by the time taken (in minutes). Walking speed (meters per second) was measured by recording the time taken in seconds (to the nearest millisecond) to walk 2.44 m (8 feet) at a normal pace from a standing start and then dividing 2.44 m by this time. Walking aids were permitted for this test (n=88), and participants were asked to complete it twice with the fastest of the 2 speeds achieved used in analyses. Standing balance time was measured, using a stopwatch, as the longest time, up to a maximum of 30 seconds, for which participants could maintain a 1-legged stance in a standard position with their eyes closed. Balance times were skewed and so were transformed by natural logarithm for analyses after adding 1 to avoid zero values. For each of the 4 tests, nurses recorded whether a study participant was unable or unwilling to perform the test and the reason for this (eg, health reasons, technical problems).

Physical performance tests were also conducted at age 60 to 64 years. These were the same measures as at age 69 years, except that the timed up and go test had been used instead of the walking speed test. To measure timed up and go, the time taken for participants to rise from an armless chair, walk 3 m, turn around, return to the chair, and sit down was recorded. The test was performed once at a normal pace, and walking aids were permitted (n=24). Timed up and go speed (meters/second) was calculated by dividing the distance walked (6 m) by the time taken (seconds).

### Markers of Composite Organ Damage and Inflammation at Age 60 to 64 Years

Fasting overnight blood samples were taken by nurses, initially processed at clinical research facility laboratories, and stored at −80°C. Frozen aliquots were transferred monthly to the MRC Human Nutrition Research laboratory in Cambridge. Analyses of cystatin C, NT-proBNP, E-selectin, and IL-6 were subsequently undertaken in a central laboratory (Table I in the online-only Data Supplement). We derived a cystatin-based estimated glomerular filtration rate, calculated using the chronic kidney disease Epidemiology Collaboration formulas dating from 2012.^25^

### Covariables

We identified the following covariables (at age 60–64 years unless otherwise specified) that have been shown to be related to physical capability and the novel biomarkers and that might therefore confound the analyses. The first set of covariables included sex and body size (height and body mass index [BMI]).^6,21,26^ The second set of covariables included measures of disease status (prevalent cardiovascular disease, diabetes mellitus, or kidney disease), cardiovascular risk (pulse pressure, total:high-density lipoprotein cholesterol ratio, hemoglobin A1c, and smoking), and lifetime socioeconomic position.^6,7,11^ Cardiovascular disease was based on self-reports of doctor-diagnosed myocardial infarction, stroke, angina, coronary artery bypass graft, or angioplasty procedures. Diabetes mellitus was based on self-reports of doctor-diagnosed diabetes mellitus that have been validated against general practitioner records. Chronic kidney disease was defined as cystatin-based estimated glomerular filtration rate <60 mL/min/1.73 m^2^ based on agreed criteria.^27^ We distinguished smokers from non- or ex-smokers. Pulse pressure (mm Hg) was calculated by taking the second of 2 measures of diastolic blood pressure from the second of 2 measures of systolic blood pressure. We used pulse pressure, rather than systolic blood pressure, as it acts as “an indirect index of large artery stiffness”^28^ and hence may be a better measure of cumulative damage and arterial aging, though we also examined the impact of using systolic blood pressure (see Additional Analyses). Plasma lipids (total cholesterol, triglycerides, and high-density lipoprotein cholesterol) were performed by modification of the standard Lipid Research Clinics Protocol using enzymatic reagents for lipid determination and the total:high-density lipoprotein cholesterol ratio derived. Socioeconomic position was based on father’s occupational class in childhood (at age 11 years or at 4 or 15 years if missing at 11 years) and own occupational class at age 53 years (or at earlier ages if missing at age 53 years), classified by the Registrar General’s classification into 6 social classes (I=highest and V=lowest). A third set of covariables, only included in additional analyses, were anxiety and depression using the conventional cut point of 5 or more symptoms over the previous 4 weeks from the 28-item General Health Questionnaire (GHQ28), self-reported bodily pain over the previous 4 weeks on a 6-point scale (none, very mild, mild, moderate, severe, very severe), use of statin medication (yes/no), use of antihypertensive medication (yes/no), carotid intima thickness measured (cIMT) in a subsample (see details elsewhere^29^), and self-reported doctor-diagnosed cardiovascular disease (CVD), kidney disease, or diabetes mellitus at age 69 years.

### Statistical Analysis

The mean level and SD or median and interquartile range of all the physical capability outcomes and the biomarkers of organ damage/stress or inflammation were derived for the total sample and separately for men and women using their original units of measurement. For subsequent analyses, the continuous biomarkers were transformed using the natural logarithm to reduce skewness and then standardized (to have a mean of 0 and a SD of 1) for comparative purposes. In preliminary analyses, we tested for nonlinearity between each standardized biomarker score at age 60 to 64 years and each outcome at age 69 years by (1) testing a quadratic function, (2) including the biomarkers split by quintiles and comparing associations between models that included the 5 groups as continuous or categorical exposures, and (3) using fractional polynomials.^30^ Using a nonlinear function did not improve the model fit except for the relationship between IL-6 and standing balance where there was some evidence that adding a quadratic term improved the fit. However, because this did not change the overall findings, we present the main tables assuming linear relationships throughout. We tested, a priori, whether the relationships between the main exposures and each outcome differed by sex by adding exposure by sex interactions.

### Modeling Strategy

We investigated the associations between cystatin C, NT-proBNP, IL-6, and E-selectin at age 60 to 64 years with each physical capability outcome at age 69 years, adjusting first for sex and then additionally for height and BMI. We then additionally adjusted for the same performance measure at age 60 to 64 years (except for walking speed, where we used timed up and go speed) to assess whether the novel biomarkers were associated with decline in performance between ages 60 to 64 years and 69 years. Finally, we further adjusted for disease status, conventional cardiovascular risk factors, and lifetime socioeconomic position for those biomarkers that remained associated with the outcomes at the 5% level to see whether the novel biomarkers were still associated conditional on these measures.

### Additional Analyses

We tested the cross-sectional associations of the novel biomarkers and the other covariables with physical capability outcomes at age 60 to 64 years in those who had repeat performance measures at age 69 years, and compared the estimates with the estimates at age 69 years to see whether they differed.

We reran the final regression models including a biomarker and disease status interaction term to investigate whether the associations were similar between those with and without disease. We also reran these regression models excluding those with CVD, diabetes mellitus, or kidney disease. In this sample, we additionally adjusted for new-onset CVD, kidney disease, or diabetes mellitus at age 69 years to see whether this explained associations between the biomarkers and outcomes. We repeated the main analyses including those unable to do the physical capability tests because of health problems. We did this by giving a value equal to the midpoint of the lowest sex-specific fifth (n=12 for grip strength, 44 for chair rise speed, and 14 for walking speed) and a value of zero for standing balance time (n=55). To check whether our findings changed, we also reran the multivariable analyses 4 more times: using systolic blood pressure rather than pulse pressure, additionally adjusting for bodily pain and caseness on the GHQ28, additionally adjusting for statin and antihypertensive medications, and taking account of subclinical vascular disease in a subsample by additionally adjusting for cIMT. STATA14 was used for all analyses.

Data used in this publication are available to bona fide researchers on request to the NSHD Data Sharing Committee via a standard application procedure; details can be found elsewhere.^31^

## Results

The maximum sample for analysis was 1736 individuals with ≥1 measure of physical capability at age 69 years and measures of cystatin C or NT-proBNP and both height and BMI at age 60 to 64 years. Table 1 shows the descriptive characteristics for the total sample and for men and women separately. Men had stronger grip, longer balance times, and faster chair rise and walking speeds at ages 60 to 64 years and 69 years. At age 60 to 64 years, levels of cystatin C and E-selectin were higher in men but levels of NT-proBNP were higher in women; there was no difference in IL-6 levels. Levels of pulse pressure and total/high-density lipoprotein cholesterol ratio were higher in men; there was no difference in levels of hemoglobin A1c. Men were taller, more likely to have CVD, diabetes mellitus, or kidney disease, and more like to have a higher adult social class; there were no sex differences in BMI or childhood social class.

Higher levels of all 4 biomarkers were associated with weaker grip strength, shorter balance times, and slower chair rise and walking speeds at age 69 years in sex-adjusted models (Table 2, model 1). The association between NT-proBNP and chair rise speed was only apparent in men (*P* value for interaction=0.002). After adjusting for height and BMI, cystatin C, NT-proBNP, and IL-6 remained inversely associated with all the outcomes, and E-selectin remained inversely associated with grip strength and chair rise speed (model 2). On further adjustment for the equivalent performance test at age 60 to 64 years, these associations were attenuated: all biomarkers remained inversely associated with grip strength, all but E-selectin remained inversely associated with standing balance time and walking speed, and NT-proBNP and IL-6 remained inversely associated with chair rise speed (model 3).

Correlations between the novel biomarkers were all modest and <0.2. Mutually adjusting for all 4 biomarkers in the same model and adjusting for sex, height, and BMI showed that E-selectin was not associated independently with any of the physical capability outcomes (Table 3) and was not considered further in the analysis. Most of the other pre-existing associations were modestly attenuated, although there were no longer associations between cystatin C and chair stands and IL-6 and standing balance.

**Figure 1.**
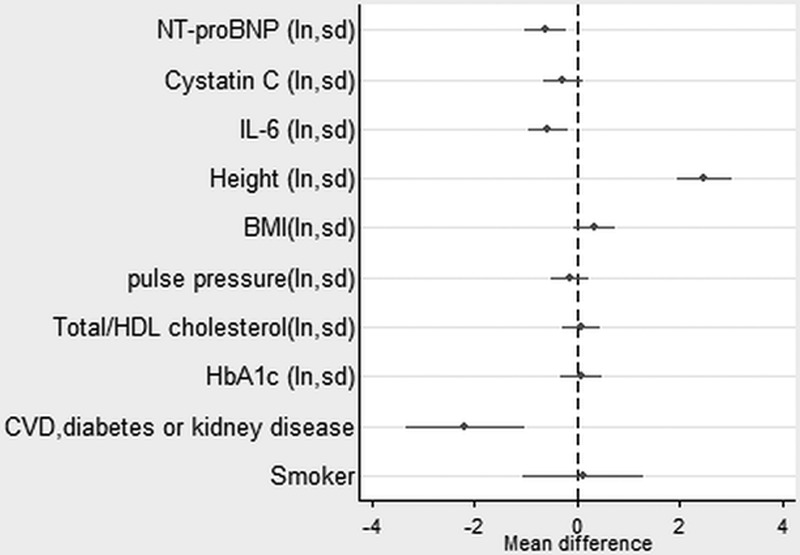
**Estimates from a linear regression model showing the mean difference in grip strength (kg) by mutually adjusted natural logged novel and conventional risk factors (also adjusted for sex and childhood and lifetime socioeconomic position).** See also Table II in the online-only Data Supplement. BMI indicates body mass index; CVD, cardiovascular disease; HbA1c, hemoglobin A1c; HDL, high-density lipoprotein; IL, interleukin; ln, natural logarithm; NT-proBNP, N-terminal pro-B-type natriuretic peptide; and sd, 1 standard deviation.

**Figure 2.**
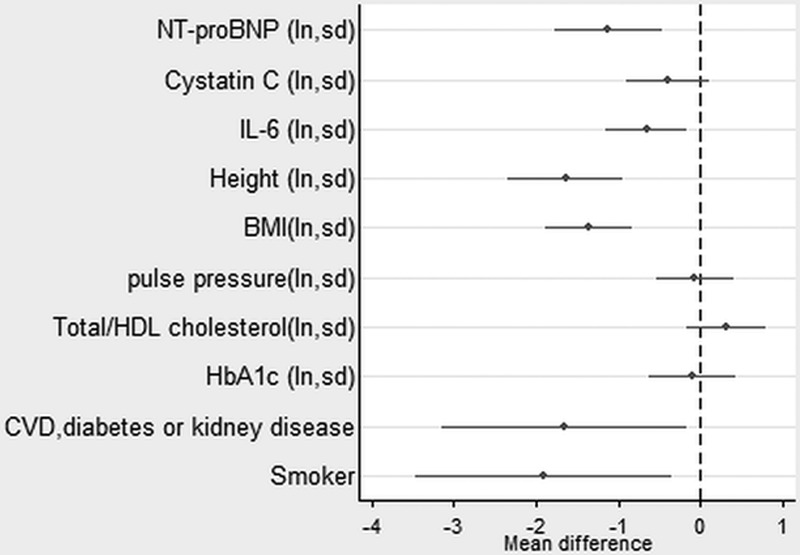
**Estimates from a linear regression model showing the mean difference in chair rise speed (stands per minute) by mutually adjusted natural logged novel and conventional risk factors (also adjusted for sex and childhood and lifetime socioeconomic position).** Estimate for NT-proBNP is for men. See also Table II in the online-only Data Supplement. BMI indicates body mass index; CVD, cardiovascular disease; HbA1c, hemoglobin A1c; HDL, high-density lipoprotein; IL, interleukin; ln, natural logarithm; NT-proBNP, N-terminal pro-B-type natriuretic peptide; and sd, 1 standard deviation.

**Figure 3.**
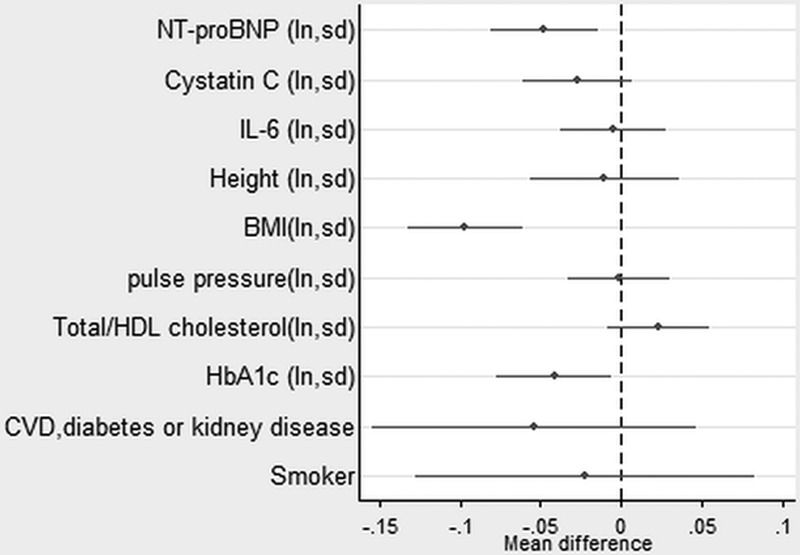
**Estimates from a linear regression model showing the mean difference in standing balance time (natural logarithm, seconds) by mutually adjusted natural logged novel and conventional risk factors (also adjusted for sex and childhood and lifetime socioeconomic position).** See also Table II in the online-only Data Supplement. BMI indicates body mass index; CVD, cardiovascular disease; HbA1c, hemoglobin A1c; HDL, high-density lipoprotein; IL, interleukin; ln, natual logarithm; NT-proBNP, N-terminal pro-B-type natriuretic peptide; and sd, 1 standard deviation.

**Figure 4.**
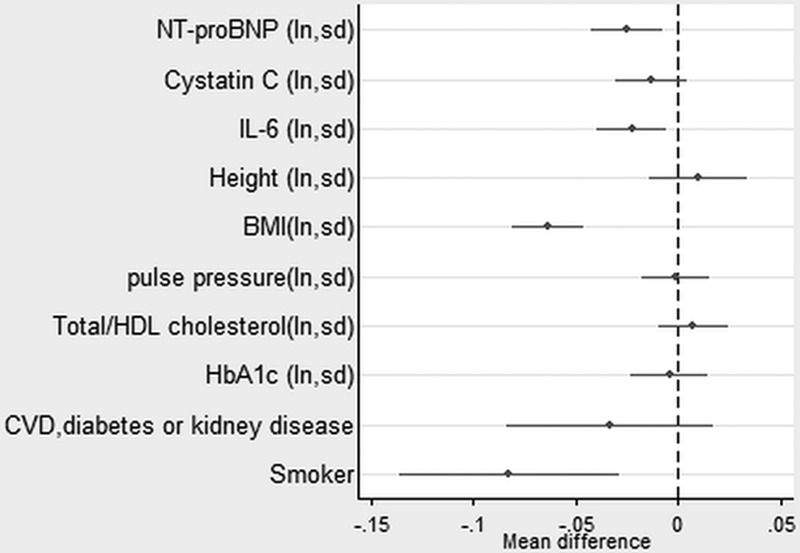
**Estimates from a linear regression model showing the mean difference in walking speed (meters per second) by mutually adjusted natural logged novel and conventional risk factors (also adjusted for sex and childhood and lifetime socioeconomic position).** See also Table II in the online-only Data Supplement. BMI indicates body mass index; CVD, cardiovascular disease; HbA1c, hemoglobin A1c; HDL, high-density lipoprotein; IL, interleukin; ln, natural logarithm; NT-proBNP, N-terminal pro-B-type natriuretic peptide; and sd, 1 standard deviation.

In the sample with complete data on covariables, higher levels of NT-proBNP continued to be strongly associated with all the outcomes (except chair rise speed in women) after taking account of disease status and conventional cardiovascular risk factors (Figures 1–4, Table II in the online-only Data Supplement). Higher levels of IL-6 remained associated with weaker grip strength and slower chair rise and walking speeds, but were no longer associated with standing balance time. Levels of cystatin C were the most strongly attenuated in the fully adjusted model and were no longer independently associated with any of the outcomes. The inverse association between NT-proBNP and chair rise speed continued to be stronger in men rather than women.

In these final models, women continued to have weaker grip strength, slower chair rise speed, and shorter balance times. Those of heavier BMI had slower chair rise and walking speeds and shorter balance times, and those of taller stature had stronger grip but slower chair rise speed. There were few associations of disease status or cardiovascular risk factors with the physical capability outcomes in the fully adjusted models. In these models, participants with CVD, diabetes mellitus, or kidney disease had weaker grip strength and slower chair rise speed. Smokers had slower chair rise and walking speeds. Those with higher levels of hemoglobin A1c had shorter balance times, and those of lower adult socioeconomic position had slower walking speed.

### Additional Analyses

The estimates for the cross-sectional associations between the novel biomarkers and the physical capability outcomes at age 60 to 64 years were smaller or similar for NT-proBNP and E-selectin but larger or similar for cystatin C and IL-6 (except for grip strength) than the estimates with the outcomes at age 69 years (Table III in the online-only Data Supplement).

The estimates for model 2 (shown in Figures 1–4 and Table II in the online-only Data Supplement) were similar when excluding the sample with CVD, diabetes mellitus, or kidney disease (data available on demand). Within this sample, adjusting for CVD, diabetes mellitus, or kidney disease at age 69 years did not attenuate the estimates for the biomarkers (Table IV in the online-only Data Supplement). We found no evidence of an interaction between disease status and the novel biomarkers on grip strength, chair rise, or walking speeds. Nor did our findings change when substituting systolic blood pressure for pulse pressure, or including GHQ28 caseness and bodily pain (Table V in the online-only Data Supplement) or statin and antihypertensive medication (Table VI in the online-only Data Supplement). In the subsample with cIMT, estimates for the biomarkers hardly changed when cIMT was added to the final models (data available on demand).

The overall findings remained the same when including those unable to do the tests for health reasons. Estimates in the main analyses (Table 2 and Figures 1–4) generally became very slightly bigger, with the association between NT-proBNP and chair rise speed strengthened the most.

## Discussion

In a large population-based study, higher levels of NT-proBNP, a marker of cardiac damage or stress, and to a slightly lesser extent IL-6, a measure of systemic inflammation, were inversely associated with subsequent levels of grip strength, standing balance, chair rise speed (men only), and walking speed over a 6-year follow-up during the seventh decade of life. Both these biomarkers were associated with these outcomes after adjusting for the equivalent outcome at baseline, suggesting that they were related to a faster decline in performance. Notably, the associations per 1-SD change in log NT-proBNP with most of these outcomes were generally much stronger than associations with similar increments in lipids, blood pressure, or glycemia and 5 times as strong as smoking status. The magnitude of effect of a 1-SD increase in log NT-proBNP (0.63 kg grip strength in the adjusted model) was equivalent to a 1.17-year decrease in grip strength (average decline, 0.54 kg per year). Higher levels of cystatin C were also associated with the decline in grip strength, chair stands, and walking speed; however, cystatin C was not independently related to these outcomes once disease status and conventional cardiovascular risk factors were accounted for. E-selectin, considered a marker of vascular function, did not add value as it was not associated with decline nor independently associated with the outcomes once the associations between the other novel biomarkers and physical capability outcomes were taken into account.

Our results are consistent with some of the previous literature on cystatin C, IL-6, and NT-proBNP, although there is far less evidence for NT-proBNP. Cystatin C is strongly associated with both cardiovascular and noncardiovascular mortality.^32^ Elevated cystatin C, but not creatinine-based measures of renal function, was also associated with frailty and slower walking speed.^33^ The only study that has looked at functional trajectories was from the Cardiovascular Health Study and showed that cystatin and IL-6 were both associated with decline independent of other biomarkers for gait speed and grip strength but only cystatin C was associated with worsening cognitive function.^16^ Similar cross-sectional associations were seen in the InChianti study between IL-6 and grip strength, walk speed, balance, and chair stands.^34^ These cross-sectional associations were also seen in the MacArthur Studies of Successful Aging, but no associations were found for IL-6 prospectively, though higher IL-6 was associated with increased mortality.^35^ The Health ABC study also found inverse associations with IL-6 and grip strength and 400-m walk time but as part of a factor-analysis derived component with C-reactive protein and plasminogen activator inhibitor-1.^36^ These findings broadly implicate low-grade inflammation as relevant to a decline in physical capability.

Importantly, the absolute levels of NT-proBNP we observe in the NSHD fall generally within the expected range, broadly consistent with many other general cohort studies of similar age.^18^ Although NT-proBNP is well recognized as a predictor of CVD events and CVD mortality as seen in recent meta-analyses, including work co-led from some of our own group,^18–20^ very little evidence relates to its role in relation to physical capacity. In longitudinal studies of the oldest old, elevated NT-proBNP predicted worsening performance on activities of daily living and cognitive decline (Leiden 85+ study),^37^ and left ventricular mass predicted functional decline in terms of activities of daily living (Jerusalem longitudinal study).^38^ Additionally, higher NT-proBNP was associated with incident disability.^39^ NT-proBNP is an important biomarker in the diagnosis of heart failure,^40^ and the upper limit of normal for NT-proBNP is 125 pg/mL. However, low-grade elevation in NT-proBNP is related to future risk of heart failure.^18^ As such, low-grade elevation in NT-proBNP might be expected to be an early marker of end organ damage that is an antecedent of decline in physical capability. Our present study meaningfully extends existing observations and identifies the potential for future clinical use of NT-proBNP in the identification of individuals at risk of poor physical capability in later life.

We had hypothesized that biomarkers that captured cumulative adverse exposures through end-organ damage/stress (eg, NT-proBNP, cystatin C) might have stronger associations than nonspecific generic inflammatory measures. We did indeed find that the former were strongly and consistently associated, but so was IL-6, an acute phase cytokine that has been linked to greater CVD and non-CVD risks, with emerging genetic evidence for links being causal for CVD.^41^ Comparing the estimates for NT-proBNP and IL-6 suggests that the associations were similar for grip strength and walking speed but that the associations between NT-proBNP and standing balance and chair rise speed (in men) were stronger. Recently, in the Cardiovascular Health Study, a biomarker index that included both these two biomarkers was associated with mortality in older adults.^42^

Conceptually there are several reasons these types of markers may be predictive of physical capability. First, their elevations may be secondary to cardiovascular and other chronic diseases (both clinical and subclinical) and their respective risk factors, which themselves have an adverse effect on capability.^6,7^ This may explain why the effects of cystatin C in the final model were attenuated. In our final multivariable models, only grip strength was predicted by disease status at age 60 to 64 years. Disease status at age 69 years in those with no disease at age 60 to 64 years was also associated with grip strength; however, adjusting for new-onset disease had little effect on the biomarker estimates, suggesting the associations we report were not simply secondary to chronic disease. Second, they share common upstream risk factors such as adverse childhood circumstances that impact on neurodevelopmental pathways. Third, they may act beyond simple proxy markers of damage/stress but may have adverse effects or associations themselves particularly on or with neurological function. NT-proBNP has been associated with worse cognition and incident dementia, and it has been suggested that this may operate through a role in regulating the blood-brain barrier, neurotransmission, and neuroinflammation.^43,44^ Paradoxically, there is evidence that elevated NT-proBNP is associated with reduced risk of type 2 diabetes mellitus,^45^ which should be beneficial in terms of neurological damage. That said, NT-proBNP is more likely a downstream marker of cardiac or vascular stress and, given its hemodynamic actions, B-type natriuretic peptide per se is considered a protective molecule. Fourth, as these measures are captured in a continuous fashion and have been shown to add predictive value for CVD outcomes, they may add useful insights into individuals’ CVD health status and, consequently, their physical capability. Elevated cystatin C has inconsistent association with cognitive impairment, white matter hyperintensities, and dementia, with some studies showing a positive association,^46,47^ an inverse association,^48^ or a U-shaped pattern.^49^ IL-6 reflects low-grade systemic inflammation (which in turn derives from a range of other risk factors such as obesity and smoking), and there is evidence that it is on a common causal cytokine pathway for CVD and other poor health outcomes in old age.^41^ We have no good explanation for the observed sex interaction with NT-proBNP and chair rise speed and, as this was not stated a priori, it may represent a type I error.

### Strengths and Limitations

This is one of the largest population-based studies that has been able to examine a variety of biomarkers in relation to subsequent objective measures of physical capability and adjust for a wide range of potential confounders. We were able to test for linear and nonlinear function as well as sex interactions. Our findings were generally consistent both in cross-sectional and longitudinal associations, suggesting that there is no major collider bias attributable to survivorship. Another strength of this study is that the sample is in middle age and the findings suggest that early detection of those at risk of poor physical capability may be possible. Most other reports of NT-proBNP include much older populations. However, with only 2 serial measures of physical capability, we were limited in our ability to assess change. Inevitably there were missing data, but including those unable to be assessed for health reasons did not alter our findings. We acknowledge the limitation that we did not fully adjust for vascular disease, but accounting for cIMT as a surrogate for subclinical vascular disease in a subsample made no difference to our findings. Our final models may have in some cases underestimated the biomarker strength of association as a result of potential overadjustment; both novel and conventional risk factors may have bidirectional associations (eg, blood pressure effect on renal function, which in turn can modify blood pressure; early cardiac dysfunction may impact on ability to exercise, and will be associated with a rise in NT-proBNP).^50^

## Conclusions

In this large population-based study, we have found several measures of end-organ damage/stress and systemic inflammation that are associated with subsequent level and decline in physical capability. The associations with subsequent physical capability remained even after adjustment for conventional disease markers and cumulative adverse socioeconomic exposures. Novel associations between NT-proBNP and physical capability were consistent across several different measures of capability and, as such, merit further replication. Elevation of NT-proBNP may identify those in midlife at risk of accelerated physical decline. These simple and relatively cheap blood-based measures—and in the case of NT-proBNP, widely available in automated assays—could be used for risk stratification to identify individuals at risk of poor physical capability for any future targeted interventions to improve capability. Further research needs to untangle whether these associations reflect the biomarker being a good integrated measure of cumulative exposures to relevant stressors or whether they point to or reflect additional pathways, such as central nervous system dysfunction, that may mediate the associations. Randomized trials that try to reduce the rate of decline in physical capability or delay incident disability could benefit from including middle-aged adults and adding these biomarkers as intermediate outcomes.

**Table 1.**
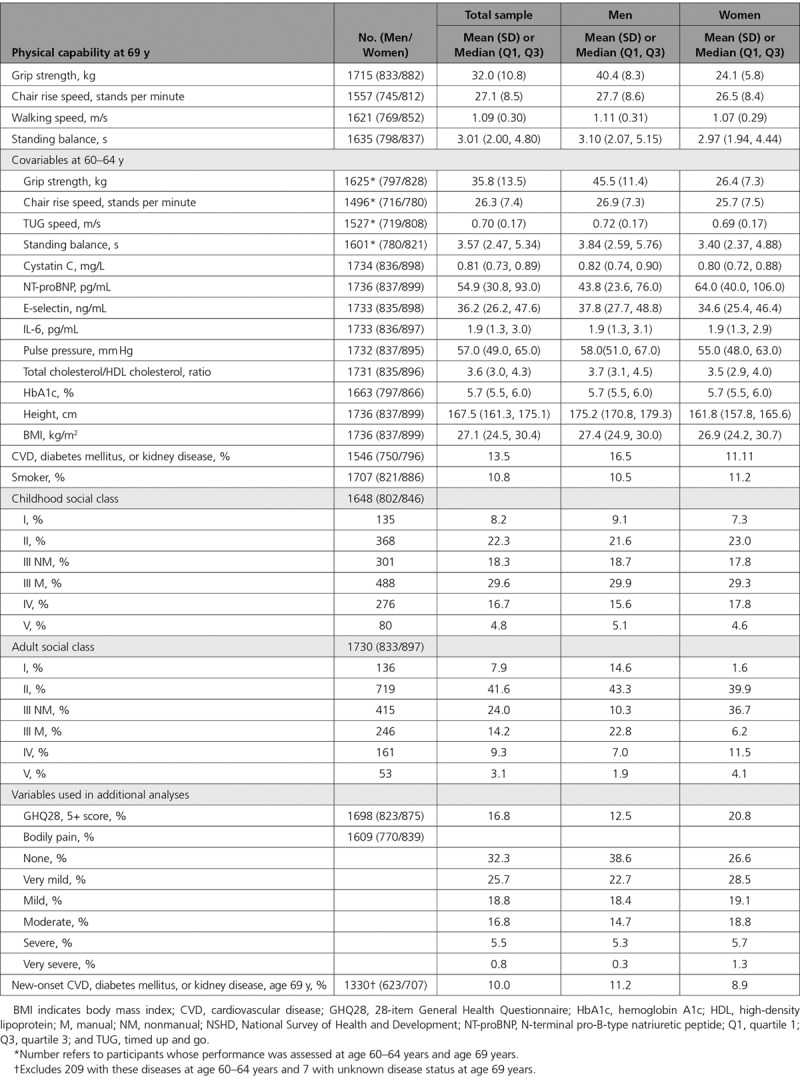
Characteristics of the Sample of 1736 (837 Men and 899 Women) Participants in NSHD With ≥1 Measure of Physical Capability at Age 69 Years and a Measure of Cystatin C or NT-proBNP, and Height and BMI at Age 60 to 64 Years

**Table 2.**
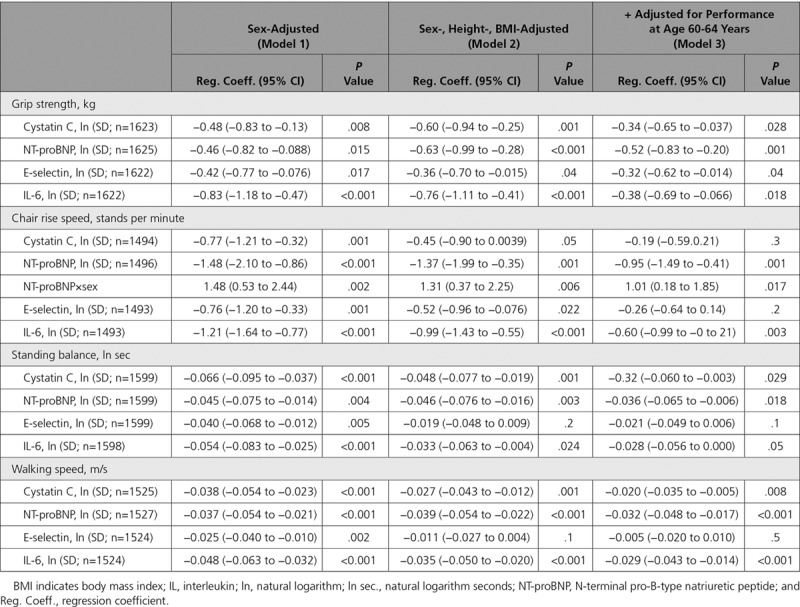
Estimates From Linear Regression Models Showing Measures of Physical Performance at Age 69 Years by 1 SD of Natural Logged Novel Biomarker, Sex-Adjusted, Then Additionally Adjusted for Height and BMI, Then Additionally Adjusted for the Same Performance Test at Age 60 to 64 Years

**Table 3.**
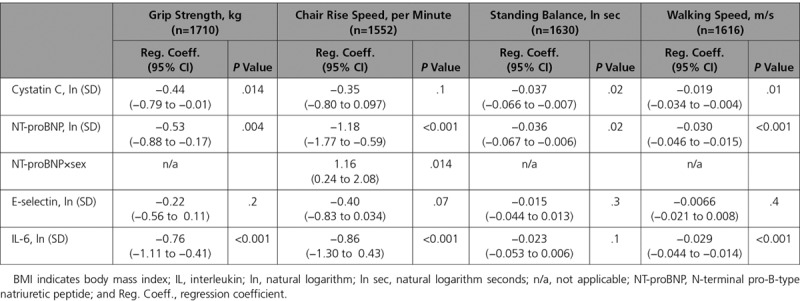
Estimates From Linear Regression Models Showing Measures of Physical Performance at Age 69 Years by Natural Logged Novel Biomarkers at Age 60 to 64 Years Mutually Adjusted and Additionally Adjusted for Sex and Standardized Height and BMI at Age 60 to 64 Years

## Acknowledgments

We thank NSHD study members for their lifelong participation and past and present members of the NSHD study team who helped to collect the data.

## Sources of Funding

This work was supported by the UK MRC MC_UU_12019/1, which provides core funding for the MRC NSHD and supports Drs Kuh, Cooper, and Hardy with MC_UU_12019/1, MC_UU_12019/2, MC_UU_12019/4. Dr Ben-Shlomo is supported by the University of Bristol. Drs Welsh and Sattar hold a separate research grant from the Chief Scientist Office (Scottish Government Health and Social Care Directorates) relating to the use of cardiac biomarkers in cardiovascular disease risk prediction (ASM/14/1). The funders had no role in the study or the decision to submit the paper for publication.

## Disclosures

All authors have completed the International Committee of Medical Journal Editors uniform disclosure form. Drs Kuh, Cooper, and Hardy report financial support from the UK MRC for the submitted work, and no financial relationships with any organizations that might have an interest in the submitted work in the previous 3 years. Dr Sattar reports personal fees from Amgen, personal fees from AstraZeneca, grants and personal fees from Boehringer Ingelheim, personal fees from Eli Lilly, personal fees from Janssen, personal fees from Novo Nordisk, and personal fees from Sanofi outside the submitted work. Dr Welsh reports grants from University College London during the conduct of the study and grants from the Chief Scientist Office outside the submitted work. Dr Ben-Shlomo reports no conflicts.

## Supplementary Material
