# Supplementary material for: Systemic Inflammation and Cardio-Renal Organ Damage Biomarkers in Middle Age Are Associated With Physical Capability Up to 9 Years Later: Findings From a British Birth Cohort Study
Source: Circulation. 2019 Jan 22;139(17):1988–99. doi: 10.1161/CIRCULATIONAHA.118.037332 (PMC6485301; doi:10.1161/CIRCULATIONAHA.118.037332)
Supplement: Supplementary file 1 [file cir-139-1988-s001.pdf]

SUPPLEMENTAL MATERIAL

Systemic inflammation and cardio-renal organ damage biomarkers in middle age are associated with physical capability up to nine years later: findings from a British birth cohort study

Diana Kuh[1] PhD FMedSci, Rachel Cooper[1] PhD, Naveed Sattar[2] MD, PhD, FMedSci, Paul Welsh[2] PhD, Rebecca Hardy[1] PhD, Yoav Ben-Shlomo Y[3] MB BS PhD

1 MRC Unit for Lifelong Health and Ageing, University College London, London, United Kingdom..

2 Institute of Cardiovascular and Medical Sciences, BHF Glasgow Cardiovascular Research Centre, Glasgow, United Kingdom

3 Population Health Sciences, University of Bristol, Bristol, United Kingdom

Supplemental Table 1. Methods and inter-assay coefficients of variation (CV) for cardio-metabolic risk factors assessed from blood samples

**Risk factor**	**Units**	**Assay/Method**	**CV (%)**
Cystatin C	mg/L	Roche Diagnostics (Burgess Hill, UK) c311 automated particle-enhanced immunoturbidimetric assay	Low control 2.5%<br>High control 2.4%
NT-proBNP	pg/ml	Roche Diagnostics (Burgess Hill, UK) e411 automated electrochemiluminescence immunoassay	Low control 6.5%<br>High control 3.8%
Interleukin-6 (IL6)	pg/ml	R&D Systems (Abingdon, UK) High sensitivity enzyme-linked immunosorbent assay (ELISA)	Intra-assay 6.6%<br>Inter-assay 13.7%
E-selectin	ng/ml	R&D Systems (Abingdon, UK) enzyme-linked immunosorbent assay (ELISA)	Intra-assay 4.1%<br>Inter-assay 8.8%
Total cholesterol	mmol/L	Siemens Dimension Xpand analyser. Cholesterol oxidase, couple with peroxidase	1.3% at 3.7mmol/L
High-density lipoprotein		Non-HDL cholesterol was inhibited from	1.7% at 1.14

cholesterol (HDL)	mmol/L	taking part in the cholesterol oxidase assay as above; the cholesterol assay then measures HDL cholesterol only.	mmol/L
Glycated haemoglobin (hbA1c)	%	TOSOH G7 analyser	1.42% at 5.67%

Supplemental Table 2. Estimates from linear regression models showing measures of physical performance at age 69 by natural logged novel and conventional risk factors

	Grip strength (n=1373)				Chair rises (n=1244)			
	Model 1<br>Sex, height, BMI adjusted		Model 2<br>Fully adjusted		Model 1<br>Sex, height, BMI adjusted.		Model 2<br>Fully adjusted	
	Reg. Coeff.<br>(95% CI)	p-value	Reg. Coeff.<br>(95% CI).	p-value	Reg. Coeff.<br>(95% CI)	p-value	Reg. Coeff.<br>(95% CI)	p-value
Cystatin C (ln,sd)	-0.54 (-0.92,-0.16)	.006	-0.28 (-0.69,0.10)	.1	-0.70 (-1.20,-0.21)	.005	-0.39 (-0.89,0.12)	.1
NT-proBNP (ln, sd)	-0.86 (-1.24,-0.48)	<.001	-0.61 (-1.00,-0.23)	.002	-1.43 (-2.07,-0.78)	<.001	-1.12 (-1.77,-0.47)	.001
NT-proBNP*sex	n/a		n/a		1.12 (0.11,2.13)	.03	1.14 (0.13,2.15)	.03
IL-6 (ln, sd)	-0.76 (-1.13,-0.38)	<.001	-0.56 (-0.95,-0.18)	.004	-0.97 (-1.45,-0.49)	<.001	-0.66 (-1.14,-0.17)	.008
Pulse pressure (ln, sd)	-0.18 (-0.55,0.18)	.3	-0.14 (-0.51,0.22)	.4	-0.09 (-0.57,0.38)	.7	-0.06 (-0.53,0.42)	.8
Total/HDL cholesterol (ln, sd)	0.25 (-0.12,0.61)	.2	0.09 (-0.27,0.46)	.6	0.36 (-0.12,0.84)	.1	0.30 (-0.18,0.79)	.2
HbA1c (ln sd)	-0.38 (-0.78,0.023)	.1	-0.07 (-0.33,0.48)	.7	-0.49 (-1.02,0.040)	.1	-0.03 (-0.63,0.44)	.7
CVD, diabetes or kidney disease	-2.61 (-3.65,-1.56)	<.001	-2.18 (-3.33,-1.03)	<.001	-2.56 (-3.92,-1.20)	<.001	-1.66 (-3.14,-0.18)	.03
Smoker	-0.63 (-1.77,0.51)	.3	-0.12 (-1.05,1.29)	.8	-2.88 (-4.38,-1.37)	<.001	-1.91 (-3.46,-0.35)	.02

Child SEP (per class)	-0.23 (-0.51,0.04)	.1	-0.13 (-0.41,0.15)	.4	-0.37 (-0.73,-0.02)	.039	-0.21 (0.57,0.16)	.3
Adult SEP (per class)	-0.20 (-0.49,0.10)	.2	-0.07 (-0.38,0.23)	.6	-0.37 (-0.75,0.02)	.059	-0.14 (-0.54,0.25)	.5
Height (ln sd)	2.56 (2.03,3.08)	<.001	2.48 (1.94,3.01)	<.001	-1.46 (-2.14,-0.78)	<.001	-1.64 (-2.33,-0.95)	<.001
BMI (ln, sd)	-0.02 (-0.39,0.34)	.9	0.32 (-0.07,0.72)	.1	-1.70 (-2.19,-1.22)	<.001	-1.35 (-1.87,-0.82)	<.001
Women v. Men	-12.2 (-13.3,-11.2)	<.001	-12.3 (-13.4,-11.2)	<.001	-3.09 (-4.44,-1.73)	<.001	-3.16 (-4.55,-1.76)	<.001

	Standing balance (n=1309)				Walking speed (n=1292)			
	Model 1<br>Sex, height, BMI adjusted.		Model 2<br>Fully adjusted		Model 1<br>Sex, height, BMI adjusted.		Model 2<br>Fully adjusted	
	Reg. Coeff.<br>(95% CI)	p-value	Reg. Coeff.<br>(95% CI).	p-value	Reg. Coeff.<br>(95% CI)	p-value	Reg. Coeff.(95% CI)	p-value
Cystatin C (ln, sd)	-0.043 (-0.076,-0.011)	.009	-0.027 (-0.061,0.006)	.1	-0.026 (-0.043,-0.009)	.003	-0.013 (-0.030,0.004)	.1
NT-proBNP (ln, sd)	-0.059 (-0.092,-0.027)	<.001	-0.047 (-0.081,-0.014)	.006	-0.036 (-0.053,-0.019)	<.001	-0.025 (-0.043,-0.003)	.005
IL-6 (ln, sd)	-0.024 (-0.057,0.008)	.1	-0.005 (-0.038,0.028)	.8	-0.034 (-0.051,-0.018)	<.001	-0.022 (-0.039,-0.006)	.009
Pulse pressure (ln,sd)	-0.008 (-0.040,0.023)	.6	-0.002 (-0.033,0.030)	.9	-0.002 (-0.018,0.015)	.8	-0.001 (-0.017,0.016)	.9
Total/ HDL cholesterol (lg, sd)	0.026 (-0.005,0.058)	.8	0.023 (-0.009,0.055)	.2	0.007 (-0.009,0.024)	.4	0.007 (-0.009,0.024)	.4

HbA1c (lg, sd)	-0.055 (-0.090,-0.020)	.002	-0.041 (-0.077,-0.006)	.02	-0.014 (-0.032,0.003)	.1	-0.004 (-0.023,0.014)	.6
CVD, Diabetes or kidney disease	-0.14 (-0.23,-0.050)	.003	-0.054 (-0.15,0.047)	.3	-0.069 (-0.12,-0.022)	.004	-0.033 (-0.084,0.017)	.2
Smoker	-0.005 (-0.050,0.040)	.1	-0.022 (-0.13,0.083)	.7	-0.12 (-0.17,-0.068)	<.001	-0.083 (-0.14,-0.029)	.002
Childhood SEP	-0.019 (-0.043,0.004)	.1	-0.010 (-0.034,0.014)	.4	-0.016 (-0.029,-0.004)	.009	-0.0071 (-0.020,0.005)	.3
Adult SEP	-0.040 (-0.065,-0.014)	.002	-0.029 (-0.056,0.003)	.029	-0.026 (-0.040,-0.013)	<.001	-0.018 (-0.032,-0.004)	.010
Height (lg, sd)	-0.004 (-0.049,0.041)	.9	-0.010 (-0.056,0.036)	.8	0.018 (-0.005,0.042)	.1	0.0010 (-0.014,0.034)	.4
BMI (lg, sd)	-0.11 (-0.15,-0.083)	<.001	-0.097 (-0.13,-0.062)	<.001	-0.075 (-0.091,-0.058)	<.001	-0.063 (-0.081,-0.046)	<.001
Women v. Men	-0.11 (-0.19,-0.017)	.02	-0.087 (-0.18,-0.005)	.06	-0.024 (-0.071,0.022)	.3	-0.002 (-0.071,0.025)	.3

.

Supplemental Table 3. Estimates from linear regression models showing measures of physical performance at ages 60-64 by 1SD of natural logged novel biomarkers, sex adjusted and further adjusted for height and BMI.

	Grip strength (kg)				Chair stands (per minute)			
	Sex adjusted		Sex, height, BMI adjusted		Sex adjusted		Sex, height, BMI adjusted	
	Reg. Coeff. (95% CI)	p-value	Reg. Coeff. (95% CI)	p-value	Reg. Coeff. (95% CI)	p-value	Reg. Coeff. (95% CI)	p-value
Cystatin C (n=1623/1494)	-0.50 (-0.98,-0.024)	.040	-0.72 (-1.19,-0.25)	.003	-0.73 (-1.12,-0.33)	<.001	-0.47 (-0.87,-0.073)	.021
NT-proBNP (n=1625/1496)	-0.12 (-0.62,,0.37)	.6	-0.36 (-0.84,0.12)	.1	-0.55 (-0.97,-0.13)	.010	-0.55 (-0.96,-0.13)	.009
E-selectin (n=1622/1493)	-0.14 (-0.61,0.33)	.6	-0.12 (-0.59,0.34)	.6	-0.75 (-1.13,-0.36)	<.001	-0.51 (-0.90,-0.12)	.010
IL6 (n=1622/1493)	-1.10 (-1.58,-0.62)	<.001	-1.11 (-1.58,-0.63)	.005	-0.97 (-1.35,-0.58)	<.001	-0.75 (-1.14,-0.36)	<.001
	STANDING BALANCE (ln sec)				TUG/WALKING SPEED (m. per sec)			
Cystatin C (n=1599/1525)	-0.078 (-0.10, -0.051)	<.001	-0.062 (-0.090,-0.035)	<.001	-0.020 (-0.047,-0.013)	.001	-0.015 (-0.024,-0.006)	.001
NT-proBNP (n=1601/1527)	-0.041 (-0.069,-0.012)	.006	-0.039 (-0.068,-0.011)	<.001	-0.012 (-0.022,-0.003)	.009	-0.012 (-0.022,-0.003)	.008
E-selectin (n=1599/1524)	-0.010 (-0.037,0.016)	.5	-0.007 (-0.37,0.016)	.5	-0.018 (-0.027,-0.010)	<.001	-0.013 (-0.022,-0.004)	.003
IL-6 (n=1598/1524)	-0.036 (-0.063,-0.0085)	.010	-0.020(-0.047,0.008)	.2	-0.019 (-0.028,-0.010)	<,.001	-0.014 (-0.023,-0.005)	.002

Supplemental Table 4. Estimates from linear regression models showing measures of physical performance at age 69 by natural logged novel risk factors at age 60-64 and new onset CVD, kidney disease or diabetes by age 69 for men and women with no CVD, kidney disease or diabetes at age 60-64.

	Sex, height, BMI adjusted		Fully adjusted*		Additionally adjusted for disease status at age 69	
GRIP STRENGTH (kg) (n=1175)	Reg. Coeff. (95% CI)	p-value	Reg. Coeff. (95% CI).	p-value	Reg. Coeff. (95% CI)	p-value
Disease status, age 69	-1.40 (-2.69,-0.11)	.03			-1.52 (-2.84,-0.19)	.03
Cystatin C (ln,sd)			-0.27 (-0.72,0.18)	.2	-0.26 (-0.71, 0.19)	.3
NT-proBNP (ln, sd)			-0.68 (-1.11,-0.25)	.002	-0.67 (-1.10,-0.24)	.002
IL-6 (ln, sd)			-0.56 (-0.95,-0.18)	.004	-0.46 (-0.88,-0.05)	.03
CHAIR RISE SPEED (stands per minute) (n=1071)						
Disease status, age 69	0.72 (-1.00,2.44)	.4			1.07 (-0.70,2.84)	.2
Cystatin C (ln,sd)			-0.49 (-1.07,0.093)	.1	-0.49 (-0.07,0.089)	.1
NT-proBNP (ln, sd)			-0.91 (-1.65,-0.16)	.02	-0.89 (-1.63,-0.15)	.02
NT-proBNP*sex			0.83 (-0.29,1.96)	.2	0.78 (-0.35,1.91)	.2

IL-6 (ln, sd)			-0.80 (-1.33,-0.27)	.003	-0.81 (-1.34,-0.27)	.003
STANDING BALANCE (ln sec) (n=1132)						
Disease status, age 69	0.032 (-0.083,0.15)	.6			0.062 (-0.054,0.18)	.3
Cystatin C (ln,sd)			-0.040 (-0.079,-0.002)	.04	-0.041 (-0.079,-0.0024)	.04
NT-proBNP (ln, sd)			-0.057 (-0.094,-0.020)	.002	-0.057 (-0.094,-0.020)	.002
IL-6 (ln, sd)			-0.004 (-0.040,0.032)	.8	-0.0042 (-0.040,0.031)	.8
WALKING SPEED (m. per sec) (n=1108)						
Disease status, age 69	-0.043 (-0.10,0.02)	.2			-0.035 (-0.095,0.026)	.3
Cystatin C (ln,sd)			-0.016 (-0.036,0.004)	.1	-0.016 (-0.036,0.004)	.1
NT-proBNP (ln, sd)			-0.027 (-0.046,-0.007)	.007	-0.027 (-0.046,-0.007)	.008
IL-6 (ln, sd)			-0.028 (-0.046,-0.009)	.004	-0.027 (-0.046, -0.009)	.004

***** Adjusted for sex, height, BMI, pulse pressure, tot/HDL cholesterol, HbA1c, disease status, smoking, child SEP, adult SEP

Supplemental Table 5. Estimates from linear regression models showing measures of physical performance at age 69 by natural logged novel risk factors, bodily pain and GHQ-28 caseness at age 60-64.

	Sex, height, BMI adjusted		Fully adjusted*		Additionally adjusted for bodily pain and GHQ-28 caseness	
GRIP STRENGTH (kg) (n=1330)	Reg. Coeff. (95% CI)	p-value	Reg. Coeff. (95% CI).	p-value	Reg. Coeff. (95% CI)	p-value
Bodily pain (per category)	-0.77 (-1.06,-0.47)	<.001			-0.66 (-0.96,-0.37)	<.001
GHQ-28 caseness	-0.85 (-1.85,-0.47)	.1			-0.72 (-1.70,0.26)	.2
Cystatin C (ln,sd)			-0.22 (-0.61,0.17)	.3	-0.26 (-0.61, 0.17)	.3
NT-proBNP (ln, sd)			-0.62 (-1.02,-0.23)	.002	-0.59 (-0.98,-0.20)	.003
IL-6 (ln, sd)			-0.55 (-0.94,-0.16)	.006	-0.50 (-0.89,-0.11)	.01
CHAIR RISE SPEED (stands per minute) (n=1209)						
Bodily pain (per category)	-0.96 (-1.34,-0.57)	<.001			-0.90 (1.28,-0.52)	<.001
GHQ-28 caseness	-1,72 (-3.02,-0.43)	.009			-1.52 (-2.81,-0.24)	.02
Cystatin C (ln,sd)			-0.36 (-0.87,0.14)	.2	-0.38 (-0.88,-0.13)	.1
NT-proBNP (ln, sd)			-1.06 (-1.71,-0.41)	.001	-1.04 (-1.69,-0.40)	.002
NT-proBNP*sex			1.01 (-0.10,2.02)	.05	1.12 (0.11,2.13)	.03

IL-6 (ln, sd)			-0.67 (-1.17,-0.18)	.008	-0.62 (-1.10,-0.13)	.01
STANDING BALANCE (ln sec) (n=1270)						
Bodily pain (per category)	-0.040 (-0.066,-0.014)	.002			-0.037 (-0.062,-0.011)	.005
GHQ-28 caseness	0.055 (-0.032,0.14)	.2			0.066 (-0.020,0.15)	.1
Cystatin C (ln,sd)			-0.022 (-0.057,0.011)	.2	-0.024 (-0.058,0.0095)	.2
NT-proBNP (ln, sd)			-0.044 (-0.078,-0.0099)	.01	-0.042 (-0.076,-0.0077)	.02
IL-6 (ln, sd)			-0.003 (-0.037,0.030)	.8	-0.0002 (-0.034,0.033)	.9
WALKING SPEED (m. per sec) (n=1251)						
Bodily pain (per category)	-0.039 (-0.052,-0.026)	<.001			-0.036(-0.049,-0.022)	<.001
GHQ-28 caseness	-0.018 (-0.063,0.026)	.4			-0.008 (-0.052,0.036)	.7
Cystatin C (ln,sd)			-0.014 (-0.026,0.003)	.1	-0.015 (-0.032,0.003)	.097
NT-proBNP (ln, sd)			-0.027 (-0.044,-0.009)	.003	-0.025 (-0.042,-0.007)	.006
IL-6 (ln, sd)			-0.021 (-0.037,-0.004)	.02	-0.027 (-0.046, -0.009)	.004

* Adjusted for sex, height, BMI, pulse pressure, tot/HDL cholesterol, HbA1c, disease status, smoking, child SEP, adult SEP

Supplemental Table 6. Estimates from linear regression models showing measures of physical performance at age 69 by novel risk factors, and statin and anti-hypertensive medications at age 60-64.

	Sex, height, BMI adjusted		Fully adjusted*		Additionally adjusted for medications	
GRIP STRENGTH (kg) (n=1330)	Reg. Coeff. (95% CI)	p-value	Reg. Coeff. (95% CI).	p-value	Reg. Coeff. (95% CI)	p-value
Statin medication	-0.90 (-1.90,0.10)	.1			-0.16 (-1.24,0.93)	.8
Anti-hypertensive medication	-1.14 (-2.12,-0.15)	,02			-0.60 (-1.60,0.41)	.2
Cystatin C (ln,sd)			-0.30 (-0.69,0.094)	.1	-0.28 (-0.68, 0.11)	.2
NT-proBNP (ln, sd)			-0.53 (-0.93,-0.14)	.009	-0.54 (-0.93,-0.14)	.008
IL-6 (ln, sd)			-0.59 (-0.99,-0.20)	.003	-0.59 (-0.98,-0.20)	.003
CHAIR RISE SPEED (stands per minute) (n=1204)						
Statin medication	-0.83 (-2.13,0.47)	.2			-0.11 (-1.52,1.30)	.9
Anti-hypertensive medication	-1.50 (-2.77,-0.23)	.02			-1.05 (-2.35,0.25)	.1
Cystatin C (ln,sd)			-0.38 (-0.89,0.14)	.2	-0.36 (-0.89,0.14)	.2
NT-proBNP (ln, sd)			-1.15 (-1.82,-0.48)	.001	-1.14 (-1.81,-0.47)	.001
NT-proBNP*sex			1.22 (0.19,2.25)	.02	1.18 (0.15,2.22)	.02

IL-6 (ln, sd)			-0.65 (-1.15,-0.15)	.01	-0.65 (-1.15,-0.14)	.01
STANDING BALANCE (ln sec) (n=1268)						
Statin medication	-0.059 (-0.14,0.026)	.2			-0.032 (-0.126,0.062)	.5
Anti-hypertensive medication	-0.10 (-0.19, -0.020)	.02			-0.073(-0.16,0.013)	.1
Cystatin C (ln,sd)			-0.027 (-0.060,0.007)	.1	-0.041 (-0.079,-0.002)	.04
NT-proBNP (ln, sd)			-0.048 (-0.082,-0.013)	.006	-0.048 (-0.082,-0.014)	.006
IL-6 (ln, sd)			-0.009 (-0.043,0.024)	.6	-0.009 (-0.042,0.025)	.6
WALKING SPEED (m. per sec) (n=1251)						
Statin medication	-0.013 (-0.058,0.017)	.6			0.006 (-0.042,0.054)	.8
Anti-hypertensive medication	-0.047 (-0.058,-0.031)	.03			-0.033 (-0.077,0.012)	.2
Cystatin C (ln,sd)			-0.013 (-0.030,0.0046)	.3	-0.012 (-0.029,0.006)	.2
NT-proBNP (ln, sd)			-0.026 (-0.043,-0.008)	.005	-0.025 (-0.043,-0.008)	.005
IL-6 (ln, sd)			-0.024 (-0.041,-0.007)	.006	-0.024 (-0.041, -0.007)	.006

***** Adjusted for sex, height, BMI, pulse pressure, tot/HDL cholesterol, HbA1c, disease status, smoking, child SEP, adult SEP
